# Smartphone tests quantify lower extremities dysfunction in multiple sclerosis

**DOI:** 10.3389/fneur.2024.1408224

**Published:** 2024-11-15

**Authors:** Kimberly Jin, Peter Kosa, Bibiana Bielekova

**Affiliations:** Laboratory of Clinical Immunology and Microbiology, Neuroimmunological Diseases Section, National Institute of Allergy and Infectious Diseases (NIAID), National Institutes of Health (NIH), Bethesda, MD, United States

**Keywords:** gait analysis, foot tapping, neurology, motor function, multiple sclerosis, neurological examination, smartphone app, telehealth

## Abstract

**Introduction:**

Increasing shortage of neurologists compounded by the global aging of the population have translated into suboptimal care of patients with chronic neurological diseases. While some patients might benefit from expanding telemedicine, monitoring neurological disability via telemedicine is challenging. Smartphone technologies represent an attractive tool for remote, self-administered neurological assessment. To address this need, we have developed a suite of smartphone tests, called neurological functional test suite (NeuFun-TS), designed to replicate traditional neurological examination. The aim of this study was to assess the ability of two NeuFun-TS tests—short walk and foot tapping—to quantify motor functions of lower extremities as assessed by a neurologist.

**Methods:**

A cohort of 108 multiple sclerosis (MS) patients received a full neurological examination, imaging of the brain, and completed the NeuFun-TS smartphone tests. The neurological exam was digitalized using the NeurEx^™^ platform, providing calculation of traditional disability scales, as well as quantification of lower extremities-specific disability. We assessed unilateral correlations of 28 digital biomarkers generated from the NeuFun-TS tests with disability and MRI outcomes and developed machine learning models that predict physical disability. Model performance was tested in an independent validation cohort.

**Results:**

NeuFun-TS-derived digital biomarkers correlated strongly with traditional outcomes related to gait and lower extremities functions (e.g., Spearman *ρ* > 0.8). As expected, the correlation with global disability outcomes was weaker, but still highly significant (e.g., *ρ* 0.46–0.65; *p* < 0.001 for EDSS). Digital biomarkers also correlated with semi-quantitative imaging outcomes capturing locations that can affect lower extremity functions (e.g., *ρ* ~ 0.4 for atrophy of medulla). Reliable digital outcomes with high test-retest values showed stronger correlation with disability outcomes. Combining strong, reliable digital features using machine learning resulted in models that outperformed predictive power of best individual digital biomarkers in an independent validation cohort.

**Discussion:**

NeuFun-TS tests provide reliable digital biomarkers of lower extremity motor functions.

## 1 Introduction

For patients with neurologic disorders, optimal treatment depends on timely access to neurology specialists. However, accelerated demand due to an aging US population far outpaces neurologists supply. By 2025, this national shortfall of neurologists is predicted to reach 19% (1). Strategies to reduce mismatch include shaping efficient demand, training advanced practice providers, and engaging policy and law makers (2).

Telemedicine is another popular solution. The COVID-19 pandemic has highlighted the power of telemedicine to expand healthcare, but it has also revealed certain limitations. To illustrate, a survey of neurologists in Norway revealed that providers treating multiple sclerosis (MS) or movement disorders were less satisfied with remote visits than those treating epilepsy or headaches (3). The physical nature of neurological exams makes telemedicine uniquely challenging and highlights the need for better remote assessment strategies.

Improved technology makes smartphones an attractive tool in remote neurological assessment. Indeed, multiple pharmaceutical and academic groups have developed smartphone-based tests of neurologic functions like gait and balance, such as Floodlight (4, 5), MS Sherpa^®^ (6), ElevateMS (7), and others (8–13). Data acquisition can be passive (heart rate, daily steps) or active (instructed activity, survey). While design of health-monitoring applications may be technologically straightforward, confirming their clinical value remains challenging. Indeed, the psychometric properties of tests to assess gait and posture require further development. The gold standard method generally used for the evaluation of whole-body kinematics in healthy individuals (14) and individuals with MS (15) is 3D motion analysis.

There are several approaches to smartphone-based walk analysis that previous studies have shown to be valid and reliable compared to the gold standard methodologies (16). Some investigators standardize walk time, quantifying disability through GPS distance traveled (17) or step count with smartphone-embedded algorithms (18). Others standardize the distance traveled, capturing time like the traditional timed 25-foot walk (T25FW) (9). However, a digital walking test offers the unique opportunity to extract novel digital biomarkers from triaxial accelerometers and gyroscope data built into smartphones. Such features can be extracted agnostically to human biology and successfully model clinically relevant outcomes such as fall risk (13) or distinguish patients with MS from healthy controls (19). We utilized accelerometer and gyroscope data in this study to investigate gait and posture.

To address the need for a better remote neurological examination, we developed a host of smartphone tests called the neurological functional test suite (NeuFun-TS). Unlike previously discussed applications, NeuFun-TS is designed to replicate a traditional neurological exam to quantify any motoric, cerebellar, sensory, and cognitive disability. As such, instead of general identification of abnormalities, NeuFun-TS tests map specifically to components of a traditional neurological exam. Where previous studies of NeuFun-TS tests measured motoric functions of upper extremities (20, 21) and cognition (22), this study evaluates two tests that measure motor functions of lower extremities: short walk and foot tapping.

## 2 Materials and methods

### 2.1 Participants

This study was approved by the Central Institutional Review Board of the National Institutes of Health (NIH). All participants gave written or digital informed consent in accordance with the Declaration of Helsinki.

Participants were enrolled in at least one of the following protocols: Comprehensive multimodal analysis of neuroimmunological diseases of the central nervous system (clinicaltrials.gov identifier NCT00794352) and Targeting residual activity by precision, biomarker-guided combination therapies of multiple sclerosis (TRAP-MS, NCT03109288).

**Figure 1:**
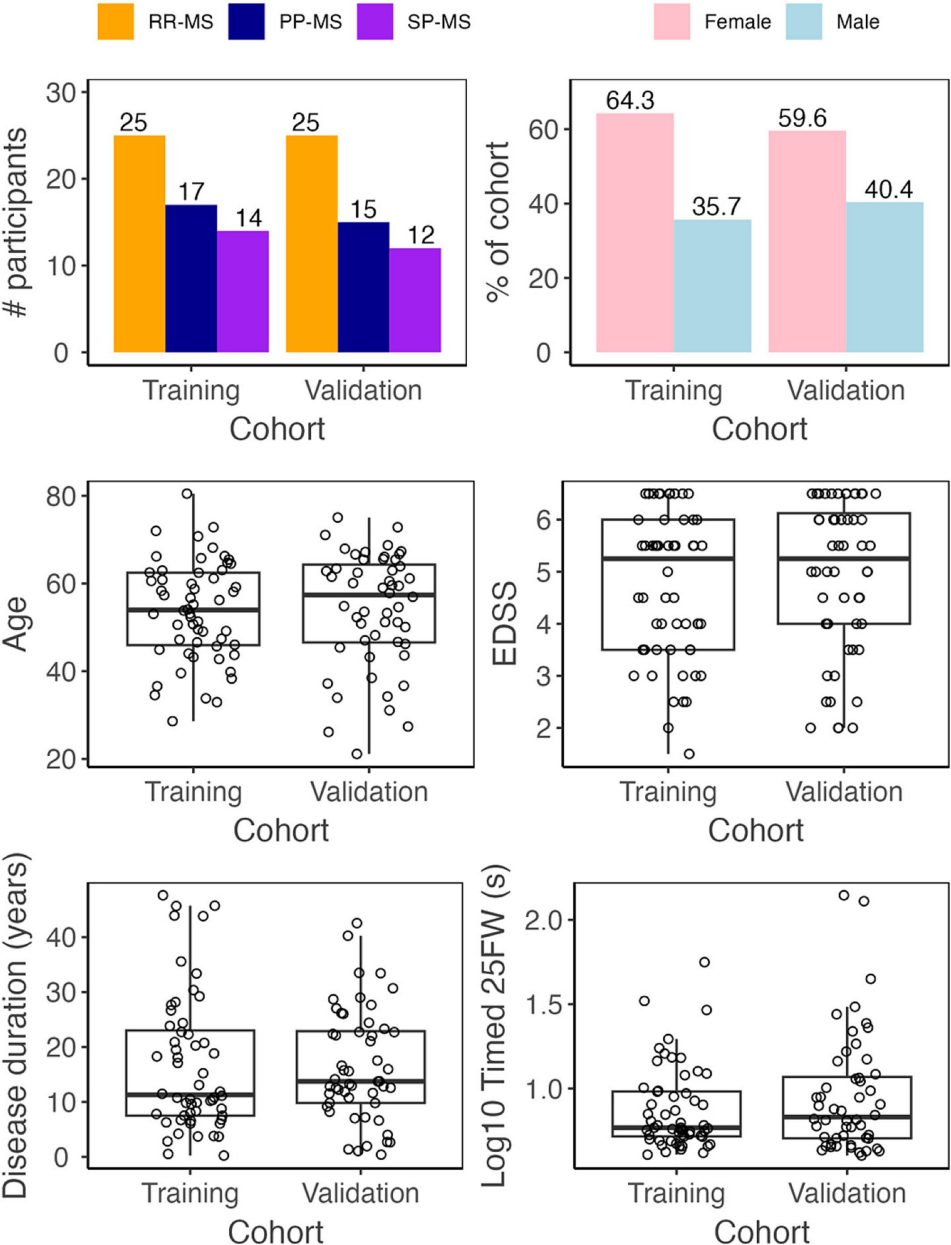
Demographic data. Before analysis, all MS patients were assigned to either a training (*N*_patient_ = 56, *N*_trial_ = 102) or validation (*N*_patient_ = 52, *N*_trial_ = 92) cohort. The two cohorts have similar diagnosis, sex, age, disease duration, EDSS, and timed 25 foot walk (25FW) distributions.

A total of 123 multiple sclerosis (MS) patients were seen between 5/1/2019 and 12/31/2021. Patients came to the NIH clinic to undergo neurological examination, brain magnetic resonance imaging (MRI), and complete NeuFun-TS smartphone tests. All participants had a diagnosis of MS based on the 2010, and later 2017, McDonald’s MS diagnostic criteria (23, 24). Seventy patients were able to walk without aid, 15 patients used unilateral support (e.g., cane), and 23 patients used bilateral support (e.g., two canes, two crutches, walker). 15 patients were unable or unwilling to complete the timed 25-foot walk or smartphone tests and were excluded. Participants were tested for COVID-19 prior their visit and no COVID-19 cases occurred during the study. Demographics and key clinical features of the remaining 108 MS patients included in this study are summarized in Table 1 and Figure 1. Fifty-two of these individuals were assigned to an independent validation cohort to evaluate models’ performance. 1:1 assignment was done randomly within MS sub-diagnosis groups: relapsing-remitting (RR-MS), primary progressive (PP-MS), and secondary progressive (SP-MS).

**Table 1:** Demographic data.

Cohort	Training	Validation
Patients (*N*)	56	52
Diagnosis (%)		
RR-MS	44.6%	48.1%
PP-MS	30.4%	28.8%
SP-MS	25.0%	23.1%
Sex (%)		
F	64.3%	59.6%
M	35.7%	40.4%
Age (years)		
Mean	53.8	54.2
SD	11.3	13
Range (min–max)	28.6–80.5	21.1–75.0
Disease duration (years)		
Mean	16.7	16.5
SD	12.5	10.1
Range (min–max)	0.2–47.6	0.4–42.6
EDSS		
Mean	4.7	4.9
SD	1.4	1.5
Range (min–max)	1.5–6.5	2.0–6.5
Timed 25 foot walk (s)		
Median	5.9	6.8
Interquartile range	4.4	6.6
Range (min–max)	4.0–56.1	4.0–140.0
Height (m)		
Mean	1.69	1.69
SD	0.09	0.11
Range (min–max)	1.50–1.86	1.42–1.89
Weight (kg)		
Mean	84.9	80.7
SD	21.0	17.2
Range (min–max)	47.3–146.1	44.7–128.0
Body mass index		
Mean	29.8	28.1
SD	6.7	5.3
Range (min–max)	16.8–46.0	17.0–39.6

*N, number; RR-MS, relapsing-remitting multiple sclerosis; PP-MS, primary progressive multiple sclerosis; SP-MS, secondary progressive multiple sclerosis; F, female; M, male; EDSS, Expanded Disability Status Scale; SD, standard deviation; min, minimum; max, maximum.*

### 2.2 Clinical outcomes

Patients underwent a full neurological examination documented into the NeurEx^™^ application that automatically computes traditional clinical outcomes used in neuroimmunology research (25), such as the Expanded Disability Status Scale (EDSS; 37), Combinatorial weight-adjusted disability score [CombiWISE (26)], and Hauser ambulation index (Hauser AI; 38). Additionally, because NeurEx^™^ digitalizes an entire neurological exam into a research database, it is easy to export quantitative data that correspond to anatomically defined systems such as cerebellar function, motor function, or sensory modality sub scores. This makes NeurEx data an excellent tool to evaluate the psychometric properties of smartphone tests. Additionally, a traditional T25FW was completed.

### 2.3 MRI outcomes

The details of acquisition and analyses of MRI data were described in detail previously (27). Briefly, MRIs were performed on 3 T Signa (General Electric, Milwaukee, WI) and 3 T Skyra (Siemens, Malvern PA) scanners equipped with standard clinical head imaging coils. T1- and T2-weighted images were reviewed by a board-certified neurologist and graded for atrophy and lesion load in cerebellum and medulla/upper cervical spine. The semi-quantitative grading levels of lesion load and atrophy consisted of “none,” “mild,” “moderate,” and “severe.” The details of grading, including visual representation of each grade, are freely available in the original publication (27).

### 2.4 NeuFun-TS tests

**Figure 2:**
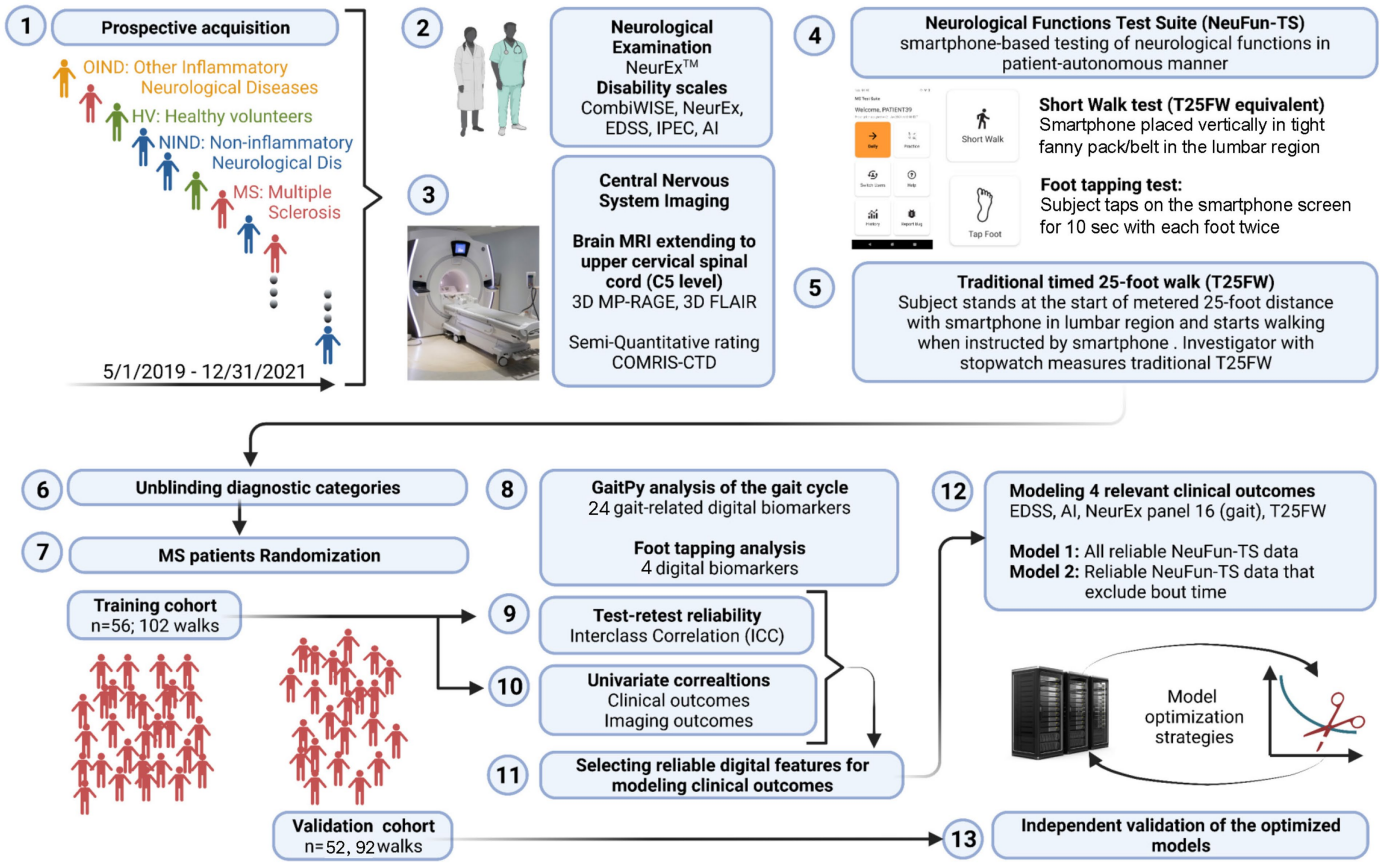
Experimental design summary. (1) From 5/2019 to 12/2021, individuals of varying diagnostic status were seen. (2) Patients underwent a full neurological examination, yielding NeurEx and disability scale scores. (3) Patients also received brain and spinal cord MRI which were rated semi-quantitatively. (4) Patients completed digital tests, including the short walk and foot tapping tests. (5) Finally, patients completed a timed 25-foot walk. (6) and (7) MS patients were assigned to either a training cohort or validation cohort. (8) Digital biomarkers were extracted from the short walk and foot tapping tests. (9) Only biomarkers with test-retest reliability were kept. (10) These reliable biomarkers were correlated against clinical and imaging outcomes. (11) and (12) These features were next used to build elastic net models of relevant clinical outcomes. (13) Finally, the performance of these models was evaluated in the independent validation cohort.

Development of any NeuFun-TS test proceeds in the following stages (Figure 2): (1) collection of NeurEx^TM^, brain MRI, and NeuFun-TS data; (2) analysis of test-rest reliability of NeuFun-TS outcomes and filtering out unreliable digital biomarkers. Entire tests may be removed given unreliable results (28); (3) assessment of univariate correlations between remaining digital biomarkers and relevant clinical and imaging outcomes; (4) aggregation of digital biomarkers to computational models of enhanced clinical value; (5) validation and quantification of the clinical value of models on a non-overlapping set of patients (independent validation cohort); (6) assessment of whether test modification may enhance usability and clinical value. Changes are guided by patient feedback and data analysis; (7) Finally, implementation of test modifications and repetition of data analysis.

The development of the NeuFun-TS short walk and foot tapping tests follow this schema. All data was collected in person at the NIH clinic. The NeuFun-TS short walk test was completed as part of the traditional T25FW. Briefly, participants placed a smartphone in a fanny pack strapped firmly to the lumbar position (L2) of the lower spine. The smartphone counted down towards a start cue, after which patients walked a pre-marked 25-foot distance as quickly and safely as possible. They were instructed to halt movement promptly after completion. A supervising investigator used a stopwatch to time the T25FW. The walk test was immediately repeated and the T25FW was averaged from two trials. Next, patients completed the foot tapping test. Briefly, patients were instructed to sit, and the smartphone was placed on a non-slip pad within comfortable reach. The smartphone counted down towards a start cue, after which patients tapped the phone screen as quickly as possible using either the toes or ball of the foot. Patients had the ability to request a repeat test if there was a technical or positioning issue. The test was completed twice, and the resulting data was averaged for analysis.

All NeuFun-TS data were directly streamed to a secure online database under alphanumeric codes. The raw values were then downloaded, processed, and analyzed in Python.

### 2.5 GaitPy data

**Figure 3:**
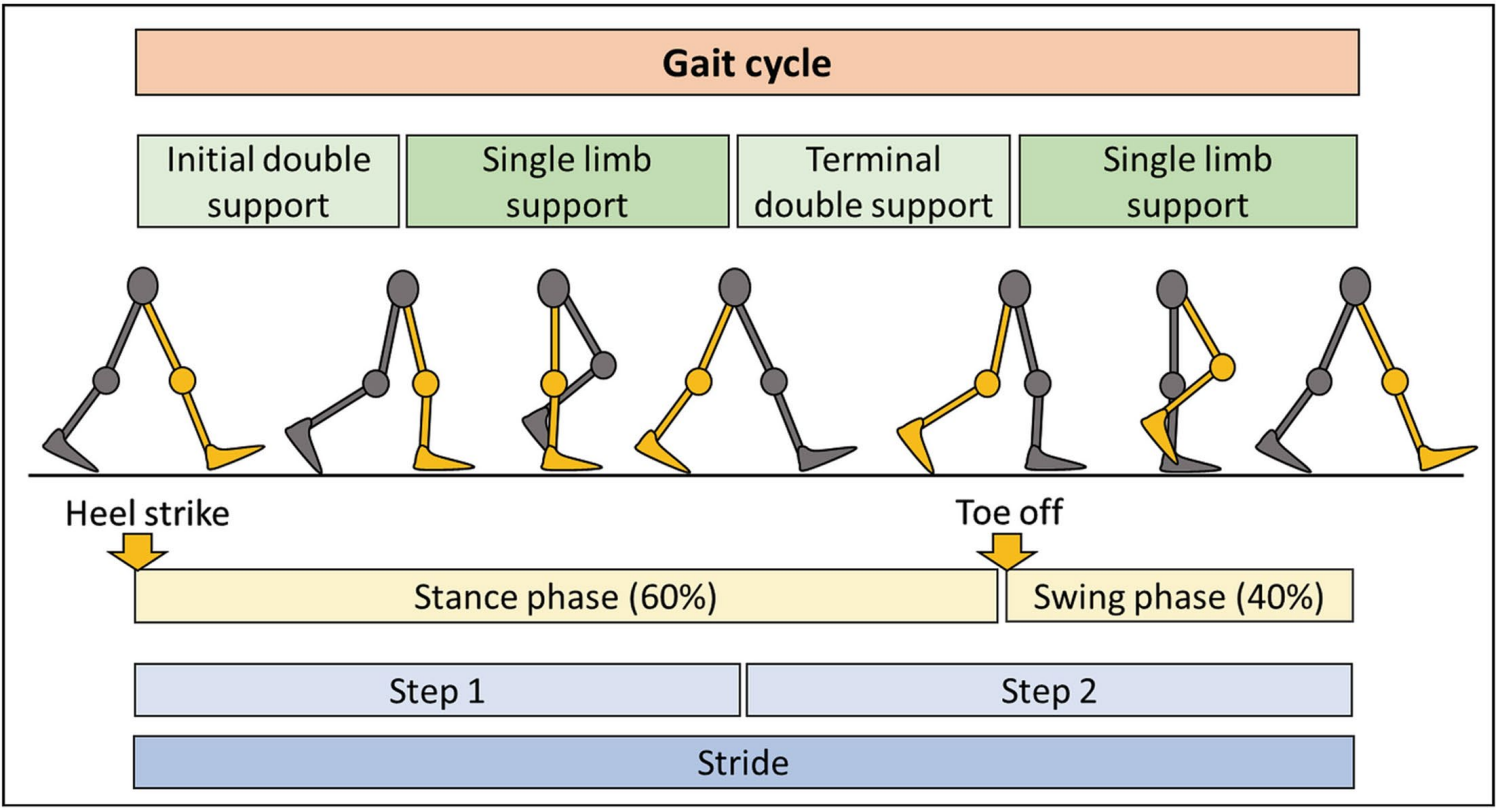
Summary of the gait cycle and terminology. The gait cycle consists of two main phases: stance phase, representing 60% of the cycle, with some contact of the foot with the ground, and the swing phase, occupying 40% of the cycle, with no contact of the foot with the ground. Each step consists of period of double and single limb support. Completion of two steps results in one stride.

We integrated accelerometer data with knowledge of the human gait cycle (Figure 3). By connecting digital biomarkers to well-characterized muscle movements, we hoped to reduce noise and identify features with high psychometric properties. Using such “domain expertise” in analyses of digital health data often strengthens clinical value of derived models (21, 22).

The open-source computational algorithm GaitPy achieves these goals by extracting gait-cycle features from vertical acceleration signal (29). It integrates elements of two previously validated algorithms: (1) Gaussian continuous wavelet transformation that removes unimportant fluctuations while amplifying gait frequency variations (30) to identify initial and final foot contacts; (2) inverted pendulum model of the body’s center of gravity to convert three-dimensional displacement of the lower trunk to gait cycle parameters (31). The cogency of these algorithms to correctly identify gait cycle features in individuals with neurological gait impairment has been previously demonstrated (32). Furthermore, previous studies have shown the validity of GaitPy algorithm compared to gold-standard gait assessment like 3D motion capture (33). See Figure 3 for a summary of gait cycle features and Table 2 for a description of GaitPy outputs.

**Table 2:** Description and reliability of raw digital biomarkers.

	Digital biomarker	Description	Spearman’s *ρ*	ICC
Reliable (Spearman’s *ρ* and ICC ≥0.75)	Gait cycles	Total # gait cycles detected	0.86^**^	0.91
	Steps	Total # steps detected	0.87^**^	0.90
	Cadence	# Steps per minute	0.88^**^	0.90
	Gait speed	Step length/step time	0.92^**^	0.90
	Step length	Average step length	0.83^**^	0.83
	Stride length	Average stride length	0.82^**^	0.82
	Step duration	Average step duration (sec)	0.91^**^	0.92
	Stride duration	Average stride duration (sec)	0.91^**^	0.90
	Stance	Average time in stance (typically 60% of cycle)	0.81^**^	0.80
	Swing	Average time in swing (typically 40% of cycle)	0.90^**^	0.75
	Initial double support	Average time in initial double support	0.81^**^	0.70
	Single limb support	Average time in single limb support	0.89^**^	0.80
	Taps/s (combined)	# Foot taps per sec (left + right average)	0.89^**^	0.90
Unreliable	Taps/s asymmetry	(Left − right foot taps)/combined foot taps	0.55^**^	0.52
	Stride duration asymmetry	Variance of all stride durations	0.43^*^	0.65
	Step duration asymmetry	Average step duration (left − right)	0.47^*^	0.45
	Step length asymmetry	Average step length (left − right)	0.14	0.02
	Stride length asymmetry	Variance of all stride lengths	0.20	0.29
	Initial double support asymmetry	Average initial double support time (left − right)	0.29	0.08
	Terminal double support	Average time spent in terminal double support	0.57^**^	0.29
	Terminal double support asym	Average terminal double support (left − right)	0.40	0.21
	Double support	Average (initial + terminal) double support	0.62^**^	0.36
	Double support asymmetry	Average double support (left − right)	0.19	0.07
	Single limb support asymmetry	Average single limb support (left − right)	0.43^*^	0.59
	Stance asymmetry	Average stance time (left − right)	0.52^**^	0.37
	Swing asymmetry	Average swing time (left − right)	0.49^*^	0.42
	Tap variance (left)	Variance of time between foot taps (left)	0.65^**^	0.19
	Tap variance (right)	Variance of time between foot taps (right)	0.78^**^	0.05

ICC, intraclass correlation coefficient, *p*-values: ^*^p < 0.05 and ^**^p < 0.005.

### 2.6 GaitPy pre-processing steps

GaitPy is optimally given several gait cycles representing steady gait. However, gait cycles at initiation and termination of walking are not reflective of steady gait, and they represent a much higher proportion of a T25FW as compared to a longer 2-6 minute walk. Subsequently, we implemented a manual quality control (QC) step to exclude initiation and termination cycles. All walk data was quality controlled together in a blinded fashion using Label Studio,^1^ an open-source data annotation tool. First, we labeled regions of fluctuating signal as “all” walking data. Within all walking data, “clean” walking data was defined as consistent, cyclic fluctuations. See Supplementary Figure S1 for a visualization of this process.

Furthermore, we hypothesized that gait parameters might be influenced by lower extremity length, which depends on height. Because height would be a confounding variable unrelated to disability, we assessed and regressed out unilateral correlations between height and GaitPy outputs. Only step and stride length required height-adjustment in this pre-processing step (Supplementary Figure S2).

### 2.7 Test-retest reproducibility

The short walk and foot tapping tests require participants to complete two trials. This allowed us to assess test-retest reproducibility using two metrics: (1) trial 1 versus trial 2 Spearman’s *ρ* (*scipy.stats.spearmanr* method) and (2) intraclass correlation coefficient (ICC) (*pingouin*.*intraclass_corr* method, ICC2, 95% confidence interval). The ICC quantifies the level of variance within an individual against variance between individuals. ICC reflects measurement reproducibility and can be interpreted according to published guidelines (34): <0.5 = “poor,” ≥0.5 but <0.75 = “moderate,” ≥0.75 but <0.9 = “good” and ≥ 0.9 = “excellent.” To focus our modeling on reproducible digital biomarkers, we defined NeuFun-TS derived biomarkers as reliable if they reached Spearman’s *ρ* ≥ 0.75 and ICC ≥ 0.75 in test-retest reliability assessment. These digital biomarkers were compared with date-matched clinical and MRI scores via a Spearman’s *ρ* correlation matrix (*pingouin.rcorr* method).

### 2.8 Aggregating functional outcomes to machine learning models of clinical value

To assess whether combination of several digital biomarkers may outperform the psychometric properties of individual ones, we chose multiple linear regression algorithms that perform both variable selection and regularization to enhance reproducibility: elastic net (EN; *sklearn.ElasticNetCV* method) and least absolute shrinkage and selection operator (Lasso; *sklearn.LassoCV* method). Although both algorithms can handle the high collinearity we observed amongst inputs (Supplementary Figure S3), we still explored collinearity reduction via principal component analysis (PCA) and exclusion of high variance inflation factor (VIF) inputs. We assessed performance of these models using 15 random 2:1 cross-validation (CV) splits of the training cohort. Based on the cross-validation results, we selected EN models for independent validation, as they achieved comparable average performance with PCA-based models but with lower performance variance (Supplementary Table S1).

The final EN models were trained on the full training cohort. The performance of these models was evaluated in the non-overlapping validation cohort consisting of patients whose data did not contribute in any way to the development or optimization of models. Ultimately, 26 models were validated, so we utilized a stricter Bonferroni-corrected significance value of *p* ≤ 0.001 to consider validation results statistically significant.

## 3 Results

### 3.1 Test-retest reliability of digital biomarkers assessing lower extremity neurological functions

Each participant completed the short walk and foot tapping NeuFun-TS tests twice, which allowed calculation of test-retest variance for all extracted biomarkers. As described in the methods section, we defined “reliable” digital biomarkers as those where trial 1 and 2 reached Spearman’s *ρ* ≥ 0.75 and ICC ≥0.75 (Table 2).

Six walk (number of steps and gait cycles, cadence, step and stride duration, and gait speed) and one foot tapping biomarkers [number of foot taps per second (left + right average)] achieved an excellent reproducibility with an ICC ≥ 0.9. An additional six walk biomarkers achieved good reproducibility with an ICC ≥ 0.75. These 13 reliable digital biomarkers were then used for modeling of outcomes.

### 3.2 Univariate correlations between digital biomarkers with relevant clinical and imaging outcomes

Recognizing that height may influence digital gait outcomes, we investigated correlations of biomarkers with height. Indeed, step and stride length had positive correlation with height (*ρ* = 0.37 and 0.40 respectively, *p* < 0.001). Subsequently, we regressed out the effects of height from both digital biomarkers (Supplementary Figure S2).

Next, we assessed correlation of reliable digital biomarkers from the short walk and foot tapping NeuFun-TS tests with relevant clinical and imaging outcomes. First, we selected traditional lower extremity-specific outcomes for comparison: traditional T25FW [in seconds (s), average of 2 trials with maximum limited to 180 s], Hauser ambulation index (Hauser AI; ordinal scale 0–9), and NeurEx gait-specific subpanel (NeurEx^™^ Panel 16; continuous scale 0–59). Next, we selected outcomes reflecting neurological disability of the entire body: EDSS (ordinal scale, 0–10), CombiWISE (continuous scale, 0–100), and NeurEx (continuous scale, theoretical maximum of 1,349). Finally, we selected semi-quantitative brain MRI outcomes that reflect central nervous system tissue injury in topographic locations that can affect lower extremities functions, such as level of atrophy and T2 lesion load in the medulla/upper cervical spinal cord and the cerebellum. The clinical value of these semi-quantitative outcomes was previously validated (27).

**Figure 4:**
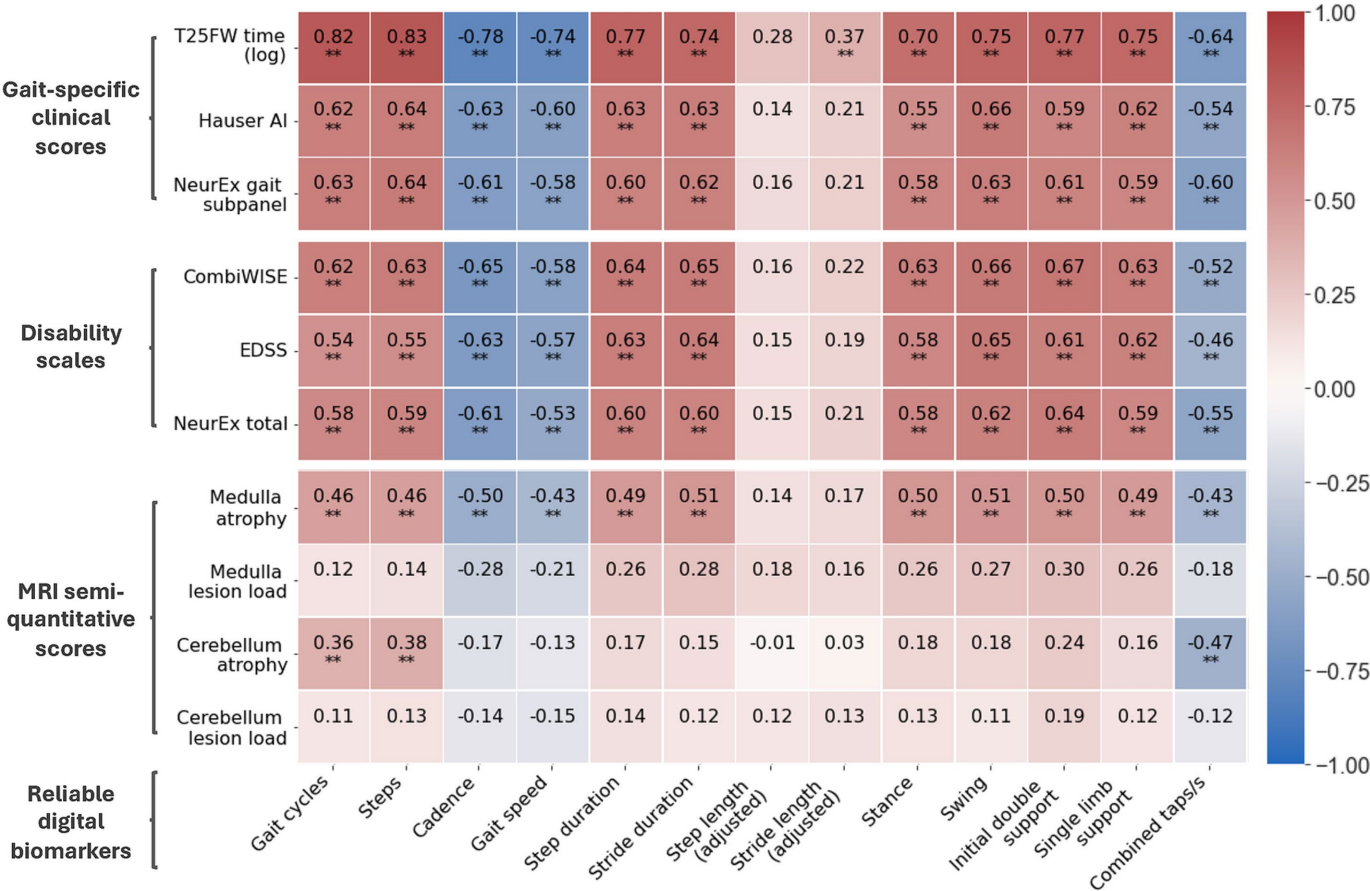
Correlation matrix of reliable digital biomarkers with date-matched clinical/MRI scores. Along with digital biomarkers extracted from the short walk and foot tapping tests, patients have date-matched MRI, disability scale, and gait-specific clinical scores. The Spearman’s *ρ* of reliable digital biomarkers and these scores are shown. All data is from the training cohort (*N*_patient_ = 56, *N*_trial_ = 102). ^*^p ≤ 0.01 and ^**^p ≤ 0.001.

Digital biomarkers with the highest ICCs also had stronger correlations with clinical outcomes (Figure 4). Furthermore, digital biomarkers correlated with clinical outcomes (up to *ρ* = 0.82; *R*^2^ = 0.67; *p* < 0.001) more strongly than with imaging outcomes (up to *ρ* = 0.51; *R*^2^ = 0.26; *p* < 0.001). The strongest correlations were with the traditional T25FW, particularly for total time-dependent digital biomarkers like gait cycles and steps. Of the global disability scales, smartphone-derived lower extremity biomarkers correlated the strongest with CombiWISE, which is a composite scale that includes the T25FW (up to *ρ* = 0.67; *R*^2^ = 0.45; *p* < 0.001).

For imaging scores, atrophy of the medulla/upper cervical spinal cord correlated moderately with most digital biomarkers (up to *ρ* = 0.51, *R*^2^ = 0.26, *p* < 0.001) whereas atrophy of cerebellum correlated better with foot taps (*ρ* = −0.47, *R*^2^ = 0.22, *p* < 0.001) than walk biomarkers (up to *ρ* = 0.38, *R*^2^ = 0.14, *p* < 0.001). Interestingly, foot tap asymmetry had a unique, moderate correlation with cerebellar atrophy (*ρ* = 0.45, *R*^2^ = 0.20, *p* < 0.001). In contrast to atrophy, T2 lesion load in identical anatomical locations did not achieve statistically significant correlations with smartphone tests.

### 3.3 Aggregating digital biomarkers to computational models of higher clinical value, and optimizing models via exploratory cross-validation

We asked whether digital biomarkers can be aggregated into models of stronger clinical value. By design, short walk and foot tapping NeuFun-TS tests assess overlapping lower extremity functions; unsurprisingly, we observed strong collinearity between digital biomarkers. Subsequently, we selected EN regression because it generates models that handle collinearity, are interpretable, and have a lower tendency to overfit than complex machine learning algorithms (Supplementary Table S1).

**Figure 5:**
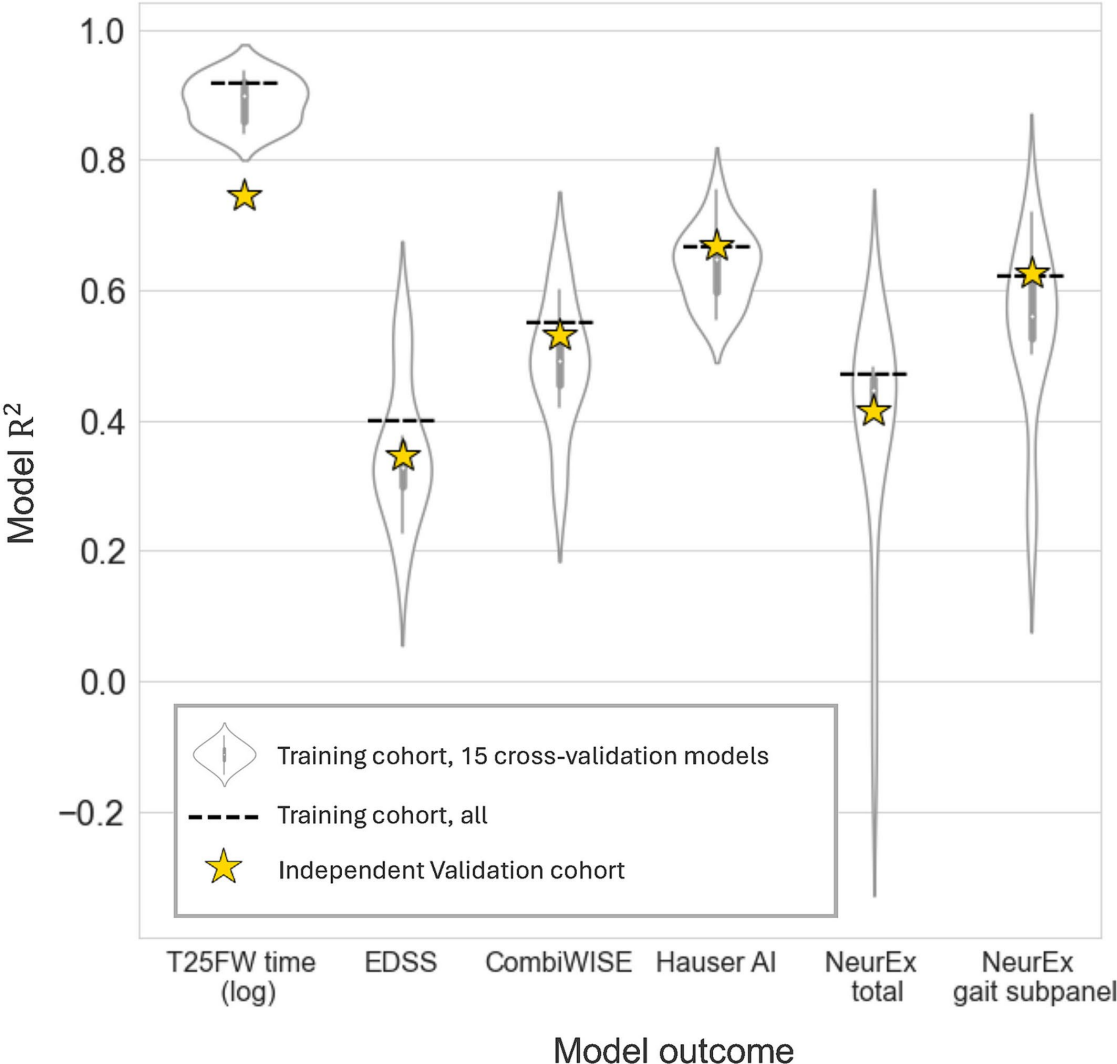
Exploratory cross-validation models and final model performance on an independent dataset. Elastic net models given all reliable digital biomarkers were generated for clinical outcomes. To explore model potential, 15 cross-validation models were trained on two thirds of the training cohort and evaluated on the remaining third (violin plots). Next, a final elastic net model was built in the entire training cohort. This model’s performance was evaluated on the data it was trained on (dashed line, *N*_patient_ = 56, *N*_trial_ = 102) and on an independent validation cohort (yellow stars, *N*_patient_ = 52, *N*_trial_ = 92).

We defined six model outcomes. The first three (EDSS, CombiWISE, and NeurEx^™^ total) capture neurological function of the entire body. The last three [T25FW time (log scale), Hauser AI, and NeurEx^™^ gait subpanel] target walking disability. We hypothesized that outcomes targeting walking disability would be better modeled by lower extremity digital biomarkers. Models were given all 13 digital features showing good test-retest reliability (Table 2). To assess the generalizability of models, we performed 15x CV within the training cohort as described in the methods section. Briefly, the training cohort was treated like its own dataset, and it was split 2:1 into a temporary CV training and CV validation cohort. A CV model was generated using the CV training data and the performance was tested on the CV validation cohort. This was repeated 15 times, and the performances are summarized by violin plots in Figure 5. While each CV model differs from the final model, the performance of these models suggests how the final model may perform on novel data. The good CV model average *R*^2^ values, particularly for T25FW (>0.8), motivated us to then generate final EN regression models using the entire training cohort. The performance of these final models in the training cohort are depicted as dashed lines in Figure 5.

### 3.4 Validation of final models in the independent cohort

Next, we evaluated the performance of these final models on an unseen set of participant data (independent validation cohort). As expected, the performance of the models in the independent validation cohort lay within the CV performance spectrum but were lower than model performance on the training cohort. Each EN model validated with Bonferroni-corrected *p* < 0.001, and the model performances are summarized by stars in Figure 5. The strongest EN model was the one predicting T25FW (*R*^2^ = 0.745), followed by models of walk-focused outcomes [Hauser AI *R*^2^ (0.669) >NeurEx^™^ gait subpanel *R*^2^ (0.626)], outperforming models of global disability outcomes [CombiWISE *R*^2^ (0.531) >NeurEx *R*^2^ (0.415) >EDSS *R*^2^ (0.346)]. Noticeably, models of CombiWISE and NeurEx, representing more granular scales with a broader dynamic range than EDSS, achieved stronger validation performance than the model of EDSS.

### 3.5 Final model features and comparison with single predictors

**Figure 6:**
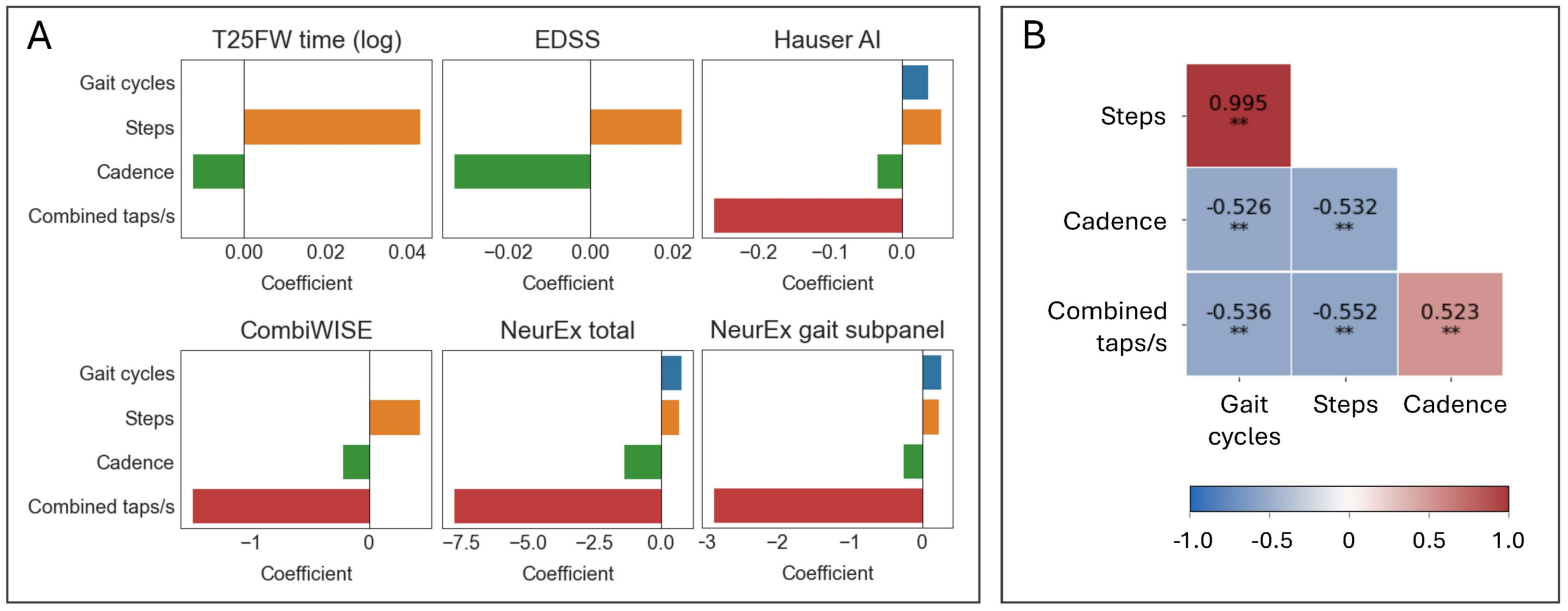
Elastic net models selected diverse features for improved prediction of clinical scores. (A) Bar graphs summarizing which features were selected by elastic net models and their coefficients. (B). A Spearman’s *ρ* correlation matrix of the selected features in the training cohort (*N* = 102). ^*^p ≤ 0.01 and ^**^p ≤ 0.001.

The final models selected up to four separate features (Figure 6A). The absolute value of a feature coefficient reflects how heavily it is weighed within a regression model, with a higher absolute value indicating more importance. While EN models of T25FW and EDSS selected only two features—steps and cadence—all other EN models also included combined taps per second and gave this predictor far greater importance. This suggests that foot taps may provide an important, non-overlapping insight into lower extremity health from gait-related inputs. This conclusion is further supported by only a moderate correlation (*ρ* between 0.52–0.55, *p* < 0.001) between combined taps per second and other predictors within the training cohort (Figure 6B). Other correlations between predictors were of a similar range (*ρ* between 0.53–0.53, *p* < 0.001), except for steps and gait cycles which were highly correlated (*ρ* = 0.995, *p* < 0.001). Despite the high collinearity between steps and gait cycles, half of the regression models selected both steps and gait cycles.

Finally, we asked whether these aggregate models predict relevant clinical outcomes better than the best single digital biomarker. Within the training cohort, we generated single predictor models (simple linear model) and the previously described final EN models. Next, we compared the *R*^2^ of these models in the validation cohort (Table 3). While all EN models showed stronger predictive value than the best single digital biomarkers, the increase in effect size for some was marginal: for example, T25FW (model *R*^2^ = 0.746, steps *R*^2^ = 0.738) and EDSS (model *R*^2^ = 0.346, cadence *R*^2^ = 0.301). For others, the gain was highly meaningful: for example, the Hauser AI EN model enhanced effect size by almost 100% (model *R*^2^ = 0.669, step duration *R*^2^ = 0.333), and the CombiWISE EN model enhanced effect size by more than 100% (model *R*^2^ = 0.531, step duration *R*^2^ = 0.254).

**Table 3:** Prediction power (*R*^2^) of the best single predictors and elastic net models.

Model outcome	Best training cohort single predictor	Validation cohort *R*^2^	
		Single predictor	Elastic net model
T25FW time (log)	Steps	0.738	0.746
EDSS	Cadence	0.301	0.346
CombiWISE	Step duration	0.254	0.531
Hauser AI	Step duration	0.333	0.669
NeurEx total	Cadence	0.306	0.415
NeurEx gait subpanel	Cadence	0.444	0.626

T25FW, timed 25-foot walk; EDSS, Expanded Disability Status Scale; CombiWISE, combinatorial weight-adjusted disability score, AI, ambulation index, NeurEx, neurological exam score. All *R*^2^ are significant with Bonferroni adjusted *p* < 0.001.

## 4 Discussion

### 4.1 Patient autonomous digital biomarkers show test-retest reliability and correlate with granular clinical outcomes

To be of value to patients, a smartphone health test must be both reliable and clinically meaningful. We identified digitally derived biomarkers with domain specificity that were consistent across trials, indicating good reliability (Table 2). Furthermore, taken individually, these reliable digital biomarkers correlated with not only gait-specific clinical scores but also global disability scales and MRI semi-quantitative scores of regional (medulla/upper cervical spine and cerebellum) central nervous system (CNS) atrophy (Figure 4). Given the relative simplicity of a smartphone short walk and foot tapping task, these digitized tests can provide clinically relevant insight into much less accessible scores.

### 4.2 Combining test results can enhance models of neurological health

To explore whether combining biomarkers provided any additional insight, we generated EN models of clinically useful scores. Despite high collinearity across input biomarkers, these models selected up to four different biomarkers (Figure 6). Models selecting both short walk-derived biomarkers (gait cycles, steps, cadence) and foot tapping biomarkers (combined taps/s) exhibited the greatest increases in prediction over single predictors (Table 3). We originally hypothesized that the short walk and foot tapping task supply non-overlapping insight into lower extremity neurological health, and the observed model improvement supports the value of combinatorial models. These results also indicate potential benefits to combining lower extremity test results with other digital tests results, such as finger strength and dexterity.

### 4.3 Contributions to existing literature

To address the challenges of remote neurological examination, we developed NeuFun-TS to provide detailed and clinically meaningful insight into neurological health. Other smartphone tests of neurological function have previously demonstrated meaningful outcomes, such as diagnostic group differences, correlations with patient reported quality of life scores, or various imaging biomarkers (35). This study goes a step further to leverage CNS imaging, comprehensive clinical exam data, and gait-specific subscores to assess the psychometric properties of digital biomarkers. Furthermore, previous studies focus solely on walking data, but we combined results from both a walk and tap test. The improved performance of models incorporating combined foot taps/s show how aggregating digital biomarkers can greatly enhance clinical value. Finally, we rigorously validated our models in an independent validation cohort and found them reliable; this ensures we did not overestimate the value of digital biomarkers and models. Independent validation is critically important, as only 8% of published studies predicting MS clinical features employed independent validation, which generally yields lower scores than cross-validation (36). Significantly, we did not find any smartphone-derived gait assessment showing independent validation. Taken together, the granular clinical data, integration of two lower extremity tests, and careful validation make this study’s results a strong contribution to literature showing that smartphone-derived lower extremity biomarkers can provided clinically meaningful value to patients and providers.

### 4.4 A digital T25FW is a non-optimal walk test

We originally designed the NeuFun-TS short walk test to replace the investigator-administered T25FW, which serves as an essential outcome collected longitudinally in MS clinical trials. However, this study reveals various limitations of a smartphone test emulating the T25FW: (1) it requires marking of a 25-foot distance, which complicates self-administration outside of a clinical environment; (2) self-determined start and end were often imprecise, and manual QC was required to identify true walk start and end; (3) the test is too short for minimally disabled individuals. To illustrate, a tall, athletic patient may cover the 25-foot distance with as few of 5 steps in 3 s. The potential of certain gait cycle parameters is lost due to limited number of cycles to calculate them from; (4) on the other hand, the test is too long for highly disabled patients. While able to walk, these individuals may fail to cover 25-feet in under 3 minutes. Important clinical information is lost when patients unable to walk are scored the same as those who need longer than 3 minutes.

As discussed in the methods section, the development of NeuFun-TS involves data-driven revision and reassessment of individual tests for ease of use and clinical utility. Previously discussed limitations, only appreciated during data analysis, motivates test modification. Future iterations of the NeuFun-TS short walk test will eliminate the need for distance in favor of a 2 minute time limit. Instead of manually removing initiation and termination cycles, the first and last 10 seconds of the test can be disregarded in analysis. Furthermore, a set time ensures individuals with no or little disability generate enough gait cycles for analysis, while those with moderate or worse disability can complete the test.

### 4.5 Study limitations and future directions

Non-optimal test design limits our walk data quality, potentially causing underestimation of discussed digital biomarkers to capture lower extremity health. For example, step length asymmetry showed poor test-retest reliability, but this could be due to insufficient gait cycles available to calculate asymmetry as opposed to it being a truly non-meaningful biomarker. Additionally, due to COVID-19 restrictions, we were unable to bring healthy volunteers to the NIH clinical center over the study duration. As such, we were unable to complete group comparisons and assess the physiological effects of aging on digital biomarkers. Future directions include redesign and evaluation of a 2 minute walk test and integration with other NeuFun-TS tests (21, 22).

## Data Availability

The original contributions presented in the study are included in the article/Supplementary material, further inquiries can be directed to the corresponding author.

## Footnotes

^1^ https://labelstud.io
