# Supplementary material for: Smartphone tests quantify lower extremities dysfunction in multiple sclerosis
Source: Front Neurol. 2024 Nov 15;15:1408224. doi: 10.3389/fneur.2024.1408224 (PMC11604577; doi:10.3389/fneur.2024.1408224)
Supplement: Supplementary file 2 [file Data_Sheet_2.docx]

**
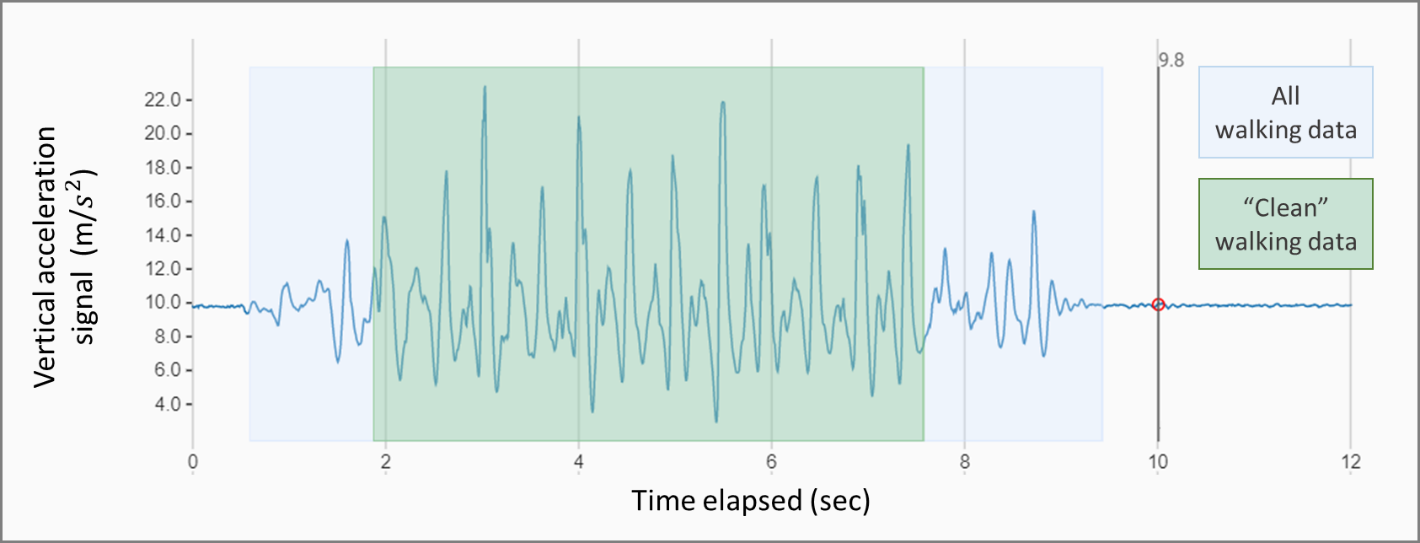
**

**Supplementary Figure S1.** To reduce noise, the central portion of walking data was labeled “Clean” and used for subsequent analyses. Clean walking data is characterized by consistent, cyclic signal suggestive of max walking velocity. An example of this quality control process is depicted here. All labels were assigned using Label Studio, an open-source data annotation tool.

**
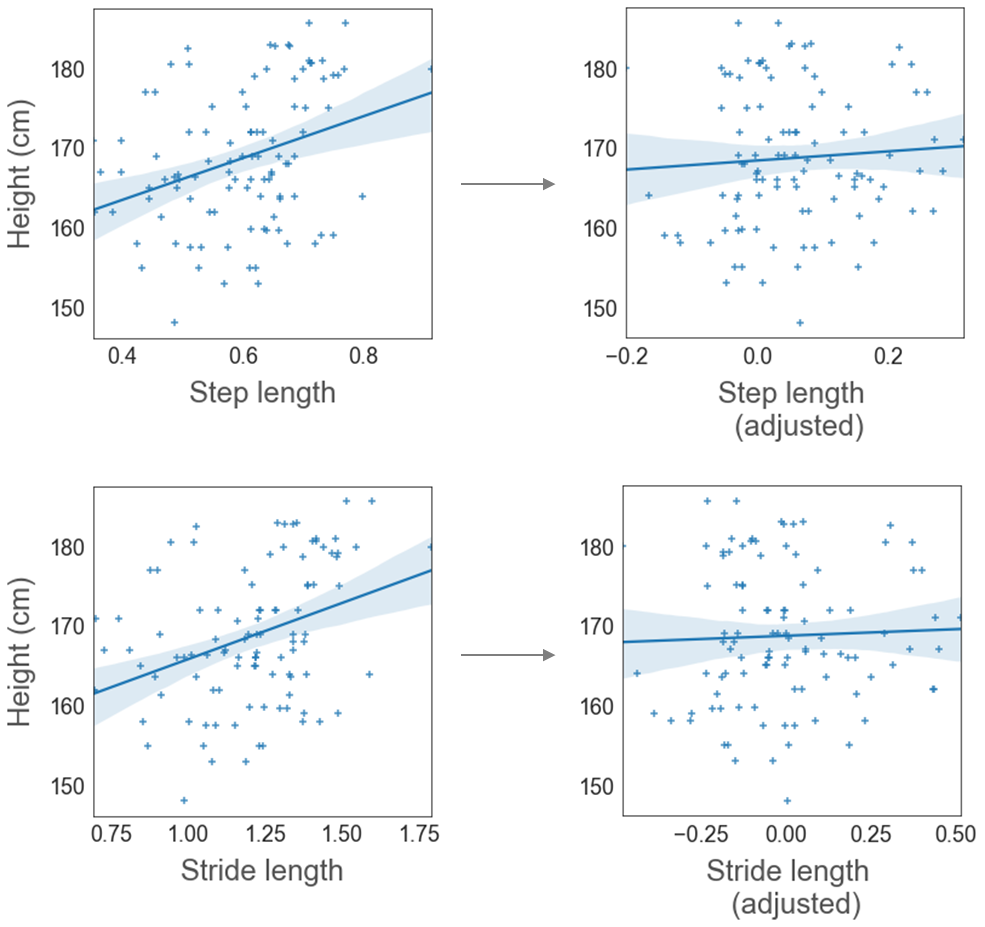
**

**Supplementary Figure S2.** The effect of height on step and stride length were regressed out before further analysis. The adjustment equation was determined within the Training cohort data (N_patient_= 56, N_trial_=102).

**
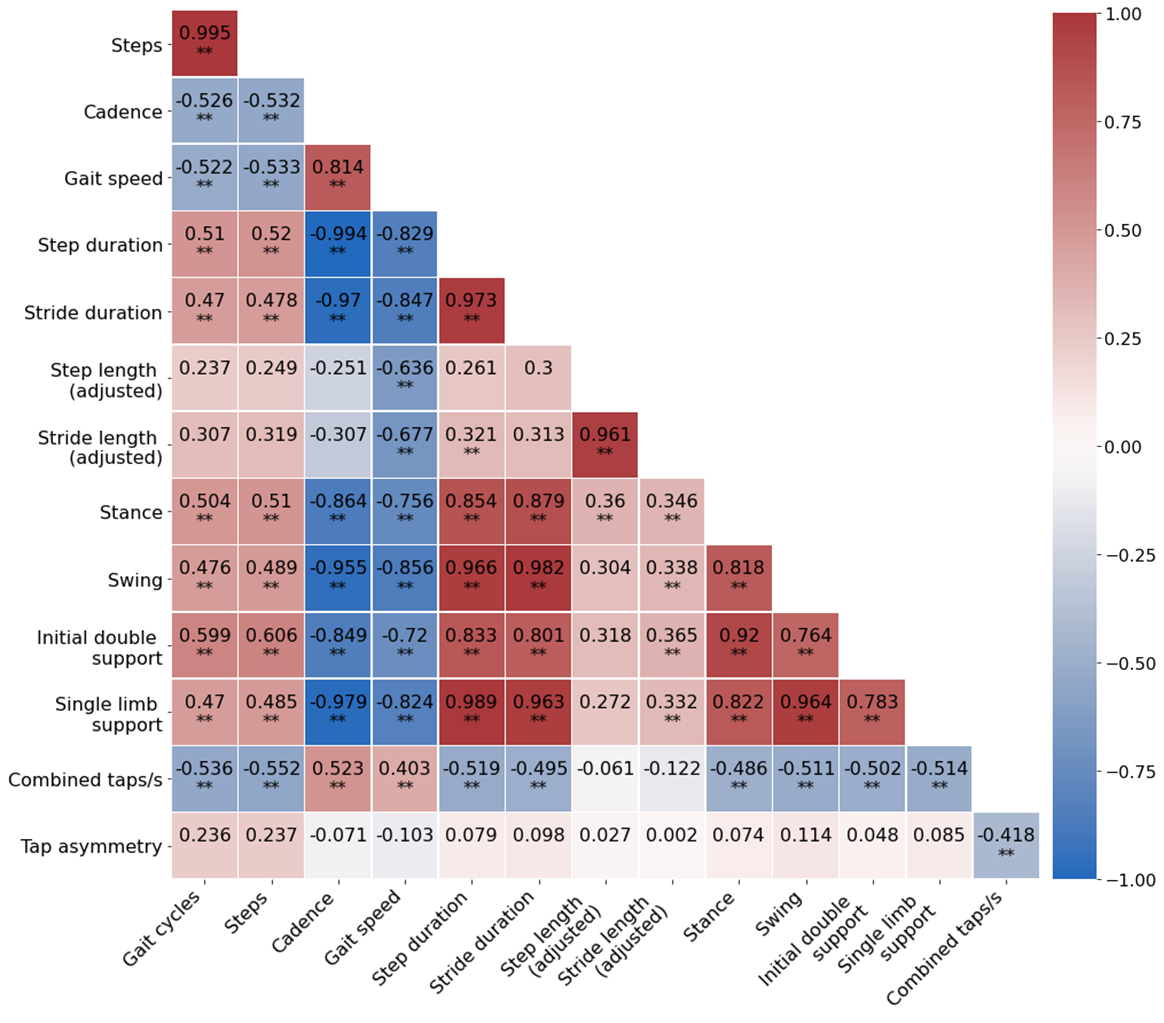
**

**Supplementary Figure S3.** Correlation matrix with Spearman’s ρ of all reliable digital biomarkers in the Training cohort (N_patient_= 56, N_trial_=102). * p-val ≤ 0.01, ** p-val ≤ 0.001

**Supplementary Table S1. Summary of 15 exploratory cross-validation models in the Training cohort.**

| **Target** | **Predictor inputs** | **Model Type** | **R^2^ Average** | **R^2^ Variance** |
| --- | --- | --- | --- | --- |
| EDSS_score | All | Lasso | 0.377 | 0.0075 |
| EDSS_score | All | EN | 0.379 | 0.0073 |
| EDSS_score | VIFs | Lasso | 0.293 | 0.0154 |
| EDSS_score | VIFs | EN | 0.295 | 0.0087 |
| EDSS_score | PCA | Lasso | 0.389 | 0.0116 |
| EDSS_score | PCA | EN | 0.404 | 0.0124 |
| CombiWISE | All | Lasso | 0.517 | 0.0061 |
| CombiWISE | All | EN | 0.528 | 0.0055 |
| CombiWISE | VIFs | Lasso | 0.456 | 0.0095 |
| CombiWISE | VIFs | EN | 0.455 | 0.0080 |
| CombiWISE | PCA | Lasso | 0.519 | 0.0246 |
| CombiWISE | PCA | EN | 0.541 | 0.0209 |
| Hauser AI | All | Lasso | 0.623 | 0.0067 |
| Hauser AI | All | EN | 0.625 | 0.0065 |
| Hauser AI | VIFs | Lasso | 0.564 | 0.0110 |
| Hauser AI | VIFs | EN | 0.558 | 0.0088 |
| Hauser AI | PCA | Lasso | 0.593 | 0.0381 |
| Hauser AI | PCA | EN | 0.606 | 0.0362 |
| NeurEx total | All | Lasso | 0.495 | 0.0063 |
| NeurEx total | All | EN | 0.488 | 0.0050 |
| NeurEx total | VIFs | Lasso | 0.433 | 0.0121 |
| NeurEx total | VIFs | EN | 0.404 | 0.0082 |
| NeurEx total | PCA | Lasso | 0.501 | 0.0293 |
| NeurEx total | PCA | EN | 0.556 | 0.0090 |
| NeurEx gait subpanel | All | Lasso | 0.619 | 0.0039 |
| NeurEx gait subpanel | All | EN | 0.624 | 0.0046 |
| NeurEx gait subpanel | VIFs | Lasso | 0.594 | 0.0110 |
| NeurEx gait subpanel | VIFs | EN | 0.552 | 0.0079 |
| NeurEx gait subpanel | PCA | Lasso | 0.634 | 0.0207 |
| NeurEx gait subpanel | PCA | EN | 0.644 | 0.0171 |

AI – ambulation index, EN – elastic net, Lasso – least absolute shrinkage and selection operator, PCA – principal component analysis, VIF – variance inflation factor
